# Are Psychiatrists Trained to Address the Mental Health Needs of Young People Transitioning From Child to Adult Services? Insights From a European Survey

**DOI:** 10.3389/fpsyt.2021.768206

**Published:** 2022-02-09

**Authors:** Frederick Russet, Veronique Humbertclaude, Nikolina Davidovic Vrljicak, Gwen C. Dieleman, Katarina Dodig-Ćurković, Tomislav Franic, Suzanne E. Gerritsen, Giovanni de Girolamo, Gaelle Hendrickx, Hala Kerbage, Fiona McNicholas, Athanasios Maras, Santosh Paramala, Moli Paul, Aurélie Schandrin, Ulrike M. E. Schulze, Cathy Street, Helena Tuomainen, Dieter Wolke, Swaran P. Singh, Sabine Tremmery, Diane Purper-Ouakil

**Affiliations:** ^1^CHU Montpellier-Saint Eloi, Médecine Psychologique de l'Enfant et de l'Adolescent, Montpellier, France; ^2^Department of Child and Adolescent Psychiatry, School of Medicine, University of Split, Split, Croatia; ^3^Department of Child and Adolescent Psychiatry and Psychology, Erasmus University Medical Center, Rotterdam, Netherlands; ^4^University of Osijek, Osijek, Croatia; ^5^IRCCS Instituto Centro San Giovanni di Dio Fatebenefratelli, Brescia, Italy; ^6^Department of Neurosciences, Child and Adolescent Psychiatry, University of Leuven, Leuven, Belgium; ^7^INSERM CESP U1018, Paris, France; ^8^Department of Child and Adolescent Psychiatry, School of Medicine and Medical Science and Geary Institute, University College Dublin, Dublin, Ireland; ^9^Yulius Academy, Rotterdam, Netherlands; ^10^Department of Child and Adolescent Psychiatry, Erasmus Medical Center-Sophia Children's Hospital, Rotterdam, Netherlands; ^11^Department of Child and Adolescent Psychiatry, Institute of Psychiatry, Psychology and Neuroscience, King's College London, London, United Kingdom; ^12^Centre for Interventional Paediatric Psychopharmacology and Rare Diseases (CIPPRD), National and Specialist Child and Adolescent Mental Health Services, Maudsley Hospital, London, United Kingdom; ^13^HealthTracker Ltd., Gillingham, United Kingdom; ^14^Warwick Medical School, Department of Psychology and Division of Mental Health and Wellbeing University of Warwick, Coventry, United Kingdom; ^15^Stratford Child and Adolescent Health Service, Coventry and Warwickshire Partnership Trust, Stratford on Avon, United Kingdom; ^16^Department of Adult Psychiatry, Nimes University Hospital, University of Montpellier, Nîmes, France; ^17^Department of Child and Adolescent Psychiatry/Psychotherapy, University of Ulm, Ulm, Germany; ^18^Department of Child and Adolescent Psychiatry, University Hospitals Leuven, Leuven, Belgium

**Keywords:** transition, transitional care, training, psychiatry, general adult psychiatry, adolescent psychiatry, child psychiatry, Europe

## Abstract

**Background:**

In mental health, transition refers to the pathway of young people from child and adolescent to adult services. Training of mental health psychiatrists on transition-related topics offers the opportunity to improve clinical practice and experiences of young people reaching the upper age limit of child and adolescent care.

**Methods:**

National psychiatrist's organizations or experts from 21 European countries were surveyed 1/ to describe the status of transition in adult psychiatry (AP) and child and adolescent psychiatry (CAP) postgraduate training in Europe; 2/ to explore the amount of cross-training between both specialties. This survey was a part of the MILESTONE project aiming to study and improve the transition process of young people at the service boundary.

**Results:**

Transition was a mandatory topic in the AP curriculum of 1/19 countries (5%) and in the CAP curriculum of 4/17 countries (24%). Most topics relevant for transition planning were addressed during AP training in 7/17 countries (41%) to 10/17 countries (59%), and during CAP training in 9/11 countries (82%) to 13/13 countries (100%). Depending on the training models, theoretical education in CAP was mandatory during AP training in 94% (15/16) to 100% of the countries (3/3); and in AP during CAP training in 81% (13/16) to 100% of the countries (3/3). Placements were mandatory in CAP during AP training in 67% (2/3) to 71% of the countries (12/17); and in AP during CAP training in 87% (13/15) to 100% of the countries (3/3).

**Discussion and Conclusion:**

Specific training about transition is limited during CAP and AP postgraduate training in Europe. Cross-training between both specialties offers a basis for improved communication between child and adult services but efforts should be sustained in practical training. Recommendations are provided to foster further development and meet the specific needs of young people transitioning to adult services.

## Introduction

In mental health care, transition refers to the pathway of a young person from a child and adolescent mental health service (CAMHS) to an adult mental health service (AMHS). This health services transition occurs at a particularly vulnerable developmental stage, when young people are also simultaneously going through social, family and academic life transitions (1, 2). Discontinuity of care may therefore have harmful consequences for those with ongoing mental health needs in various life domains (3). Unfortunately, only a minority experience good transitional care (4, 5), mostly because of a limited co-ordination between services and the lack of effective evidence-based interventions in transitional care (6, 7). Health services and policy makers from countries around the world are increasingly recognizing the importance of improving transitions from CAMHS to AMHS and developing effective interventions and models of care that are youth-informed and oriented (6, 8, 9). The European Union of Medical Specialists-Child and Adolescent Psychiatry (UEMS-CAP) is currently working on European guidelines for Transitional care (see www.uemscap.eu/working-group).

An optimal transition involves a coordinated, purposeful, planned and patient-centered process, ideally involving a period of parallel care/joint work between services, to ensure a smooth transition (5, 10, 11). Previous qualitative research showed that young people transitioning between services perceive the consistency of clinicians and periods of parallel care as being among key factors enabling effective CAMHS to AMHS transition (12, 13). This highlights the importance of having clinicians in both services trained in navigating the various aspects of transitional care.

Training of mental health professionals on transition-related topics could therefore facilitate the process at the clinical level and improve clinical practice. Among existing studies on psychiatry training across Europe (14–22), only one focused on transitional care and revealed that, according to trainees, practical and theoretical training on this topic occurred, respectively, in only 27.8 and 16.7% of European countries (21). A systematic review of psychiatrist's training in Europe highlighted that a training specifically dedicated to transition was included only in the United Kingdom (UK) and in Ireland (23). The existing literature points toward gaps in teaching about the process and planning of transition, calling for proper training in “transition psychiatry” in the postgraduate curriculum (24).

Improving knowledge about clinical presentations of mental disorders in adolescence and specific communication skills are other possible areas of training. Some mental health professionals may not feel comfortable or lack the expertise and interviewing techniques needed to work with young people and their families (25–28). Only 17% of Adult Psychiatry (AP) trainees and 35% of General Psychiatry trainees have lectures on adolescent psychopathology, vs. 61% of Child and Adolescent Psychiatry (CAP) trainees (21).

Furthermore, the transition literature shows clinical and conceptual differences between child and adult mental health service models that may lead to mutual misperceptions (4, 29) and may impede the coordination and collaborative working between services that is needed. Training content and training models may account for the cultural divide existing between CAMHS and AMHS, characterized by different beliefs and attitudes, a lack of understanding of different service structures and different working practices related to communication and information transfer (27). In Europe, trainees in AP and CAP follow a separate specialized training from the start of postgraduate studies in 55% countries (21), but there are no studies on the impact of training models on transitional care.

One of the aims of the “MILESTONE project,” a EU-funded research programme exploring the transition of young people from CAMHS to AMHS in eight countries (www.milestone-transitionstudy.eu/) (30), is to study the training of professionals in transitional care. This survey has been designed in the context of the MILESTONE project to describe the current status of transition and related issues in AP and CAP postgraduate training in Europe. It also examines the respective content of AP and CAP postgraduate training, with a particular focus on the amount of theoretical and practical cross training between both specialties (e.g., training in CAP in the AP or General Psychiatry curriculum, and vice-versa).

## Methods

Data collection was carried out between 2015 and 2018. This research did not require ethical approval as no personal data were collected.

### Development and Design of the Questionnaire

For this study, we used a 114-item questionnaire covering topics identified through a systematic review of the literature (23) and through the recommendations of the European Union of Medical Specialists (UEMS) and UEMS-CAP (31–35) (see Supplementary Material 1 for a synthesis). We verified the relevance and clarity of questions by sharing the questionnaire with members of the MILESTONE consortium. In the final version of the questionnaire (Supplementary Material 2), section 1 covered general aspects of psychiatry postgraduate training; section 2 focused on training in AP and section 3 focused on training in CAP. Specific questions addressed transition and related issues such as the developmental course of childhood disorders, adolescence-onset disorders and therapeutic issues (36).

All respondents to the survey were asked if arrangements in their country were likely to change soon. Those indicating this might be the case were recontacted at the end of the data collection period to check on any recent developments. In case of discrepancies between former and new information provided by the respondent, only the more recent one was included in the analysis.

### Recruitment

Participant recruitment was based on purposeful sampling constructed to specifically target key informants. The relevant lead of each national psychiatrist's association from 39 European countries were contacted by the research team: Albania, Austria, Belgium, Bosnia, Bulgaria, Czech Republic, Cyprus, Croatia, Denmark, Estonia, Finland, France, Germany, Greece, Hungary, Ireland, Iceland, Italy, Latvia, Lithuania, Luxembourg, Malta, Moldavia, Montenegro, Netherlands, Norway, Poland, Portugal, Romania, Russia, Serbia, Slovakia, Slovenia, Spain, Sweden, Switzerland, Turkey, Ukraine, and the United Kingdom (UK).

When official representatives were not available or declined participation, snowball sampling was used to contact university professors recommended by the national association or known to professionals involved in the MILESTONE Project.

Participants could answer using online or paper versions.

### Data Analysis

Data analysis is descriptive in nature, with missing data or inconsistent answers excluded by country for the corresponding questions.

## Results

**Figure 1:**
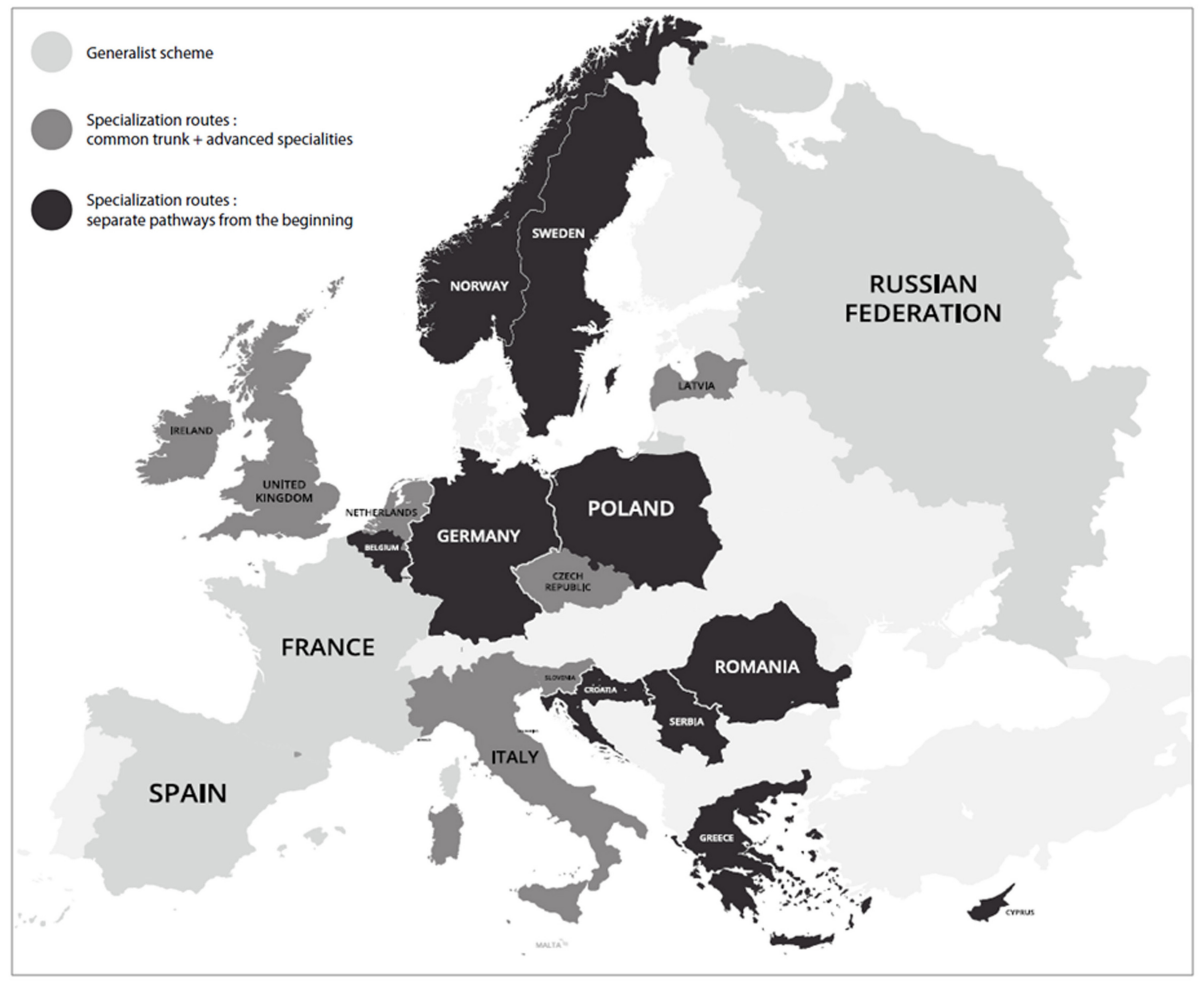
Mapping of the 3 training models.

We received completed questionnaires for 21/39 countries (54%) (Figure 1). Since the psychiatry postgraduate training in Luxembourg replicated the French model, it was excluded from the analysis. Among respondents, 50% were representatives of national psychiatry associations while 50% were from universities or research institutions engaged in training.

Eight countries indicated plans to revise their training syllabus. By study completion, updated information was available from four of them, while the change was awaiting approval in one country and had not yet occurred in another one. The remaining two countries did not provide additional information.

Three ***models of training*** in CAP and AP training were identified. In the first model, trainees are provided with a general education in psychiatry including a training in both AP and CAP, with optional subspecialities (3/20 countries, 15%, hereafter referred to as generalist countries). In the second model, trainees follow a joint psychiatry core programme, followed by mandatory further specialization (7/20 countries, 35%). In the third model, CAP and AP are considered as independent specialities and trainees are provided with separate training from the start, with different programmes (10/20 countries, 50%). Countries with model 2 or 3 are referred to as countries with specialization route. Figure 1 depicts these three models.

### In Adult Psychiatry Training

#### Transition Training and Developmental Topics

Transition in itself is a mandatory topic in the AP curriculum only in Sweden (Table 1). However, it may be addressed through a related subject in 3/15^1^. (20%) countries, during courses of CAP in the UK and Russia, or while addressing areas of clinical governance, team working, clinical management and care planning in Ireland.

**Table 1:** Transition and related issues in AP and CAP training and in Continuing Medical Education.

**TRANSITION**	**In AP training**	**In CAP training**
Transition is a mandatory topic	1/19 (5%)^a^	4/17 (24%)
If NO, discussed elsewhere	3/15 (20%)	4/11 (36%)
Addressed in main discipline or elsewhere, mandatory or not,		
Transition is discussed through:		
Some dedicated lectures	4/5 (80%)	5/5 (100%)
Case studies in placements	4/8 (50%)	5/7 (71%)
**ISSUES RELATED TO TRANSITION**		
**Developmental psychiatry**		
Discussed in^b^: Theoretical training	4/6 (66.7%)	10/10 (100%)
Practical training	3/6 (50%)	10/10 (100%)
**Developmental course of childhood disorders (ADHD, Autism)**		
Discussed in: Theoretical training	4/5 (80%)	8/8 (100%)
Practical training	2/5 (40%)	8/8 (100%)
**Psychiatry and/or psychopathology of adolescents**		
Discussed in: Theoretical training	4/7 (57%)	8/9 (89%)
Practical training	4/7 (57%)	9/9 (100%)
**Psychometric assessment (cognitive, neuropsychological, adaptive)**		
Discussed in: Theoretical training	2/2 (100%)	7/7 (100%)
Practical training	1/2 (50%)	7/7 (100%)
**Key role of the family as a support when taking care of Adolescents**		
Discussed in: Theoretical training	3/4 (75%)	8/8 (100%)
Practical training	4/4 (100%)	8/8 (100%)
**Working with partners for an optimal care of the adolescents**		
Discussed in: Theoretical training	2/2 (100%)	2/2 (100%)
Practical training	5/6 (83%)	6/6 (100%)
**OTHER ASPECTS IN RELATION WITH TRANSITION**		
Treatments for adolescents and young adults specifically discussed in pharmacotherapy training	9/18 (50%)	11/15 (73%)
Mandatory placements in a hospital ward in charge of adolescents or young people during training	6/18 (33%)	10/15 (67%)
Training providing with a global understanding of mental health services taking care of adolescents	Understanding of CAMHS	Understanding of AMHS
	8/18 (44%)	7/15 (47%)
**ISSUES RELATED TO TRANSITION DISCUSSED IN CME?**		
Developmental psychiatry	6/17 (35%)	10/13 (77%)
Developmental course of childhood disorders (ADHD, Autism)	9/17 (53%)	9/13 (69%)
Psychiatry and/or psychopathology of adolescents	8/17 (47%)	10/13 (77%)
Psychometric assessment	4/17 (24%)	6/14 (43%)
Key role of the family as a support when taking care of Adolescents	7/17 (41%)	6/16 (38%)
Working with partners for an optimal care of the adolescent*s*	9/13 (69%)	7/13 (54%)

*AP, adult psychiatry; CAP, child and adolescent psychiatry; ADHD, attention deficit and hyperactivity disorder; CAHMS, child and adolescent health mental service; AHMS, adult health mental service; CME, continuous medical education*.

(a) *Results are expressed in number of countries/number of respondent countries (percentage)*.

(b) *whether the issue is mandatory or not in training*.

**Figure 2:**
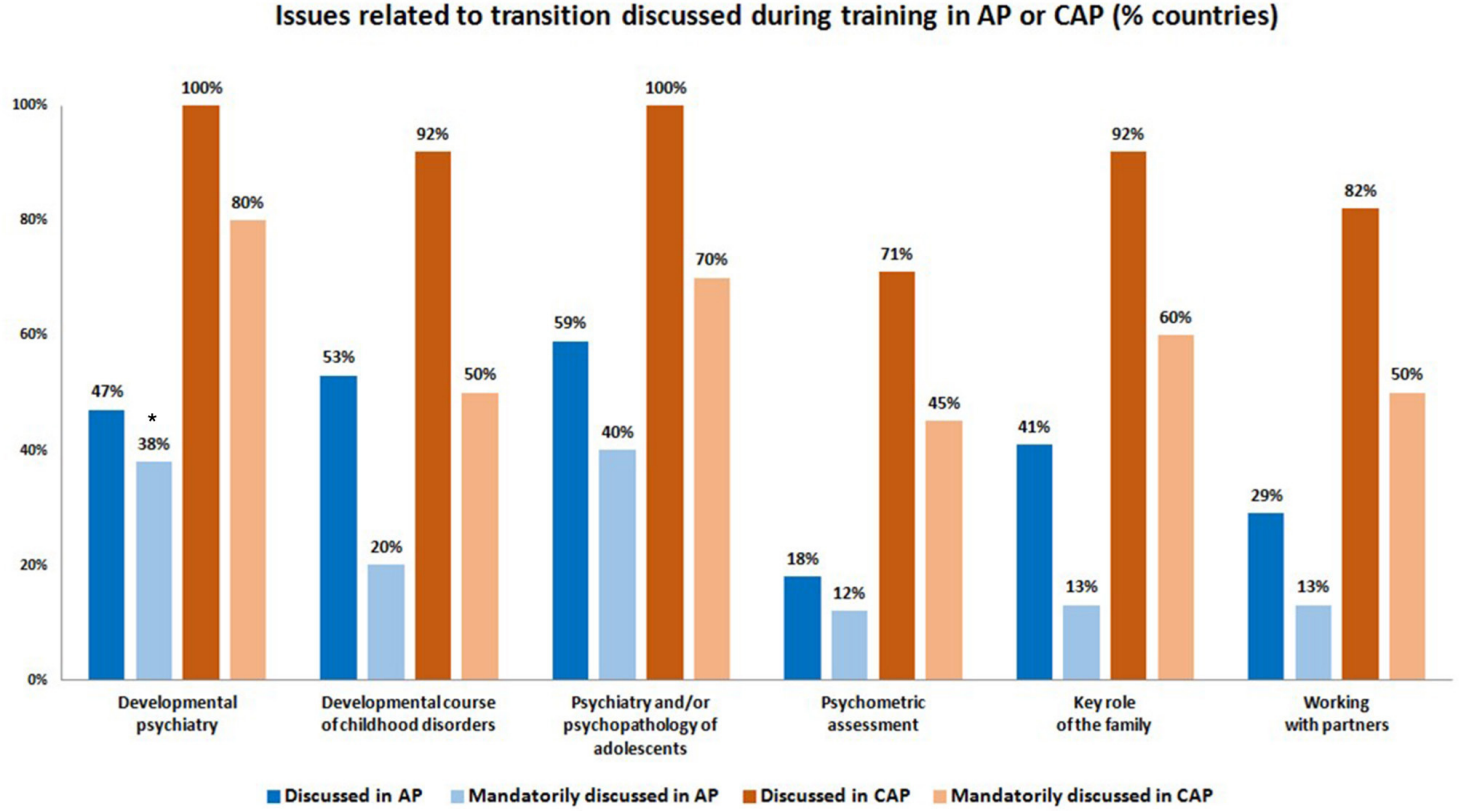
Issues related to transition discussed in AP and CAP training. (*) % mandatory = number of countries answering “yes” if an issue was *mandatorily* discussed / (number of countries responding to the question about the corresponding issue being discussed or not–number of non-responding countries to the question about the issue being *mandatorily* discussed or not).

With regard to issues related to transition in the AP curriculum, the most frequently discussed were psychiatry and/or psychopathology of adolescents (10/17 countries, 59%), developmental course of childhood disorders (9/17, 53%), and developmental psychiatry (9/17, 47%), *mandatorily* discussed in respectively 6/15 responding countries (40%), 3/15 (20%) and 6/16 (38%) (Figure 2). Key role of the family (7/17, 41%), child psychometric assessments (3/17, 18%) or working with professional partners (5/17, 29%) were less often discussed, *mandatorily* discussed in respectively 2/15 responding countries (13%), 2/17 (12%) and 2/16 (13%). In terms of post-specialization training (Continuous Medical Education), a similar pattern was observed for issues related to transition (Table 1).

Aspects related to youth and relevant to transitional care were reported to be addressed during AP training by 6/18 (33%) of respondents (e.g., mandatory placements in a hospital ward in charge of adolescents or young people) to 9/18 (50%) of respondents (e.g., treatments for adolescents and young adults specifically discussed in pharmacotherapy training) (Table 1).

#### Training Contents: Aspects of Cross-Training With CAP

Having some ***theoretical training*** in CAP along with AP theoretical education was considered mandatory in 18/19 countries (95%), more specifically in 15/16 countries with a specialization in AP (94%) and in 3/3 generalist countries (100%) (Table 2). In countries with a specialization route in AP, the content of CAP theoretical programme was different in AP education and in CAP education in 8/13 countries (62%).

**Table 2:** Training and specialization in Adult Psychiatry and Child and Adolescent Psychiatry: theoretical and practical content.

	**Training in AP**			**Training in CAP**		
**National programme**	19/19 (100%)^*^			15/19 (79%)		
**Implementation of the programme**
Nationwide	15/19 (79%)			11/14 (79%)		
Dependent on regions	1/19 (5%)			0/14 (0%)		
Dependent on Universities	1/19 (5%)			1/14 (7%)		
Mixed	2/19 (11%)			2/14 (14%)		
Compliance with the standards of the UEMS Board^**^	8.2 (4–10) [19/19 countries]***			8.3 (5–10) [14/15 countries]		
Group A^****^	6.7 (4–9) [3/3 countries]			No data available		
Groups B and C	8.5 (5–10) [16/16 countries]			8.3 (5–10) [14/15 countries]		
**Mandatory subjects in the**	**All countries**	**Group A**	**Groups B & C**	**All countries**	**Group A**	**Groups B & C**
**theoretical training programme**		**(3 countries)**	**(17 countries)**		**(3 countries)**	**(17 countries)**
AP	19/19 (100%)	3/3 (100%)	16/16 (100%)	16/19 (84%)	3/3 (100%)	13/16 (81%)
CAP	18/19 (95%)	3/3 (100%)	15/16 (94%)	20/20 (100%)	3/3 (100%)	17/17 (100%)
Old age	17/19 (89%)	1/3 (33%)	16/16 (100%)	6/18 (33%)	1/3 (33%)	5/15 (33%)
Addiction	18/19 (95%)	2/3 (67%)	16/16 (100%)	14/18 (78%)	2/3 (67%)	12/15 (80%)
Forensic	15/17 (88%)	1/3 (33%)	14/14 (100%)	12/17 (71%)	1/3 (33%)	11/14 (79%)
Emergency	17/18 (94%)	3/3 (100%)	14/15 (93%)	16/17 (94%)	3/3 (100%)	13/14 (93%)
Liaison	15/18 (83%)	1/3 (33%)	14/15 (93%)	12/17 (71%)	1/3 (33%)	11/14 (79%)
Mental handicap	4/15 (27%)	0/3 (0%)	4/12 (33%)	11/15 (73%)	0/3 (0%)	11/12 (92%)
Neurology	12/19 (63%)	1/3 (33%)	11/16 (69%)	12/18 (37%)	1/3 (33%)	11/15 (73%)
Pediatrics	1/18 (7%)	0/3 (0%)	1/15 (7%)	11/19 (58%)	0/3 (0%)	11/16 (69%)
One dominant theoretical orientation	11/20 (55%)	8/17 (47%)				
Bio psycho social	6/11 (55%)	3/8 (37.5%)				
Psychodynamics	1/11 (9%)	2/8 (25%)				
Medical	1/11 (9%)	1/8 (12.5%)				
Bio-Medical	1/11 (9%)	2/8 (25%)				
Bio-psycho social + CBT	2/11 (18%)	0/8 (0%)				
**Mandatory placements in the**	**All countries**	**Group A**	**Groups B & C**	**All countries**	**Group A**	**Groups B & C**
**practical training programme**		**(3 countries)**	**(17 countries)**		**(3 countries)**	**(17 countries)**
AP	19/19 (100%)	3/3 (100%)	16/16 (100%)	16/18 (89%)	3/3 (100%)	13/15 (87%)
CAP	14/20 (70%)	2/3 (67%)	12/17 (71%)	18/19 (95%)	2/3 (67%)	16/16 (100%)
Old age	9/17 (53%)	0/3 (0%)	9/14 (64%)	3/14 (21%)	0/3 (0%)	3/11 (27%)
Addiction	9/17 (53%)	1/3 (33%)	8/14 (57%)	7/15 (47%)	1/3 (33%)	6/12 (50%)
Forensic	7/16 (44%)	0/3 (0%)	7/13 (54%)	4/14 (29%)	0/3 (0%)	4/11 (36%)
Emergency	10/16 (63%)	2/3 (67%)	8/13 (62%)	7/13 (54%)	2/3 (67%)	5/10 (50%)
Liaison	10/17 59%)	1/3 (33%)	9/14 (64%)	7/15 (47%)	1/3 (33%)	6/12 (50%)
Mental handicap	1/14 (7%)	0/3 (0%)	1/11 (9%)	4/13 (31%)	0/3 (0%)	4/10 (40%)
‘Neurology	12/18 (67%)	1/3 (33%)	11/15 (73%)	9/16 (56%)	1/3 (33%)	8/13 (62%)
Pediatrics	2/15 (13%)	0/3 (0%)	2/12 (17%)	11/18 (61%)	0/3 (0%)	11/15 (73%)

*AP, adult psychiatry; CAP, child and adolescent psychiatry*.

(*) *Qualitative data are expressed in number of countries/number of respondent countries (percentage)*.

(**) *Likert Scale = from 1 (not at all) to 10 (completely)*.

(****) *Group A, Generalist: One curriculum only, consisting in generalist training, (and optional complementary specializations); Group B, Monospecialities: Different specialization routes, separate from the beginning; Group C, Common trunk followed by advanced specialities*.

In ***practical training*** in AP, placements in CAP were mandatory in 14/20 countries (70%), more specifically in 12/17 countries with an AP specialization (71%) and in 2/3 generalist countries (67%) (Table 2). In countries with specialization route in AP, the mean number of mandatory placements in CAP was 1, 3 (10 respondents), for a mean total clinical experience of 5, 2 months (10 respondents). In generalist countries, the mean mandatory number and total duration of placements in CAP were respectively, 2 and 12 months (1 responding country).

#### Training Contents: Aspects of Common Background With CAP

The mean level of adherence with UEMS recommendations of the AP programmes was 8.2 (range 4–10), based on a Likert scale ranging from 0 (weak) to 10 (strong) (Table 2).

Table 2 illustrates the similarities and differences of content between AP and CAP ***theoretical training*** as regards important topics for transition. Results show considerable variation in terms of whether or not important topics for transition are covered. For example, intellectual disabilities and pediatrics were little addressed in the AP curriculum, respectively in 4/15 countries (27%) and in 1/18 countries (7%). In generalist countries, intellectual disabilities or pediatrics were not listed among the mandatory topics.

Table 3 shows the differences of content between AP and CAP in the theoretical content recommended by the UEMS. During AP training, 16/23 topics were addressed in more than two-third of countries. The least commonly addressed were diversity in psychiatry (in 9/18 countries, 50%), leadership, administration, management, economics (8/16 countries, 50%), psychological tests (10/19 countries, 53%), intellectual disabilities (10/17 countries, 59%).

**Table 3:** Mandatory content of theoretical training in each speciality (according to UEMS recommendations).

	**AP**	**CAP**
Psychopathology	19/20 (95%)^*^	16/16 (100%)
Diagnosis and classification	20/20 (100%)	16/16 (100%)
Laboratory investigations	14/19 (74%)	11/15 (73%)
Developmental psychiatry	16/19 (84%)	16/16 (100%)
Intellectual disabilities	10/17 (59%)	15/16 (94%)
Psychiatric aspects of substance misuse	19/20 (95%)	14/15 (93%)
Diversity in psychiatry	9/18 (50%)	10/14 (71%)
Legal, ethical and human rights issues in psychiatry	16/19 (84%)	14/15 (93%)
Psychopharmacology	19/19 (100%)	16/16 (100%)
Multidimensional clinical management	13/19 (68%)	13/16 (81%)
Epidemiology of mental disorders	18/20 (90%)	15/16 (94%)
Forensic	17/20 (85%)	12/14 (86%)
Leadership, administration, management, economics	8/16 (50%)	8/15 (53%)
Examination of a psychiatric patient	19/20 (95%)	16/16 (100%)
Psychological tests	10/19 (53%)	12/15 (80%)
Specific disorders and syndromes	19/20 (95%)	16/16 (100%)
Old age psychiatry	19/20 (95%)	5/13 (38%)
Psychotherapies	20/20 (100%)	15/16 (94%)
Social psychiatric interventions	12/19 (63%)	11/16 (69%)
Community psychiatry	13/17 (76%)	10/15 (67%)
Research methodology	11/17 (65%)	10/14 (71%)
Psychiatric aspects of public health	12/19 (63%)	9/15 (60%)
Emergency	20/20 (100%)	13/15 (87%)

*AP, adult psychiatry; CAP, child and adolescent psychiatry*.

(*) *Results are expressed in number of countries/number of respondent countries (percentage)*.

As regards the ***practical training***, and the mandatory placements in disciplines other than CAP during AP training (Table 2), five disciplines were mandatory in association with AP training in 9/17 countries (53%) to 14/20 countries (67%). The least frequent placements relevant for transition were forensic (in 7/16 responding countries, 44%), pediatrics (2/15 countries, 13%) and intellectual disabilities (1/14 countries, 7%). We observed the same pattern when countries with a specialization in AP were considered. In generalist countries, placements in emergency care were mandatory in 2/3 countries (67%), whereas placements in addiction services, neurology and liaison were requested in 1/3 countries (33%).

#### Other Aspects of Training Contents

All countries had a national programme regarding AP training, implemented nationwide in 15/19 (79%) countries.

There was a dominant theoretical orientation in AP education in 11/20 countries (55%), mainly bio-psycho-social (55%, 6/11 responding countries) or a combination of bio-psycho-social and cognitive-behavioral (18%, 2/11).

### In Child and Adolescent Psychiatry Training

#### Transition Training and Developmental Topics

Transition was reported to be a mandatory separate topic ***in the programme of CAP*** in 4/17 countries (24%) and was addressed through another subject within the curriculum in 4 of the other 11 responding countries (36%) (Table 1).

As regards the issues related to transition in the CAP curriculum, the most frequently discussed were developmental psychiatry and psychiatry and/or psychopathology of adolescents (both in 13/13 countries, 100%), developmental course of childhood disorders and key role of the family (both in 12/13 countries, 92%), *mandatorily* discussed in respectively, 8/10 responding countries (80%), 7/10 countries (70%), 5/10 countries (50%) and 6/10 countries (60%) (Figure 2). Psychometric assessment (10/14 countries, 71%) and working with partners (9/11 countries, 82%) were less often discussed and *mandatorily* discussed in respectively, 5/11 (45%) and 5/10 (50%) responding countries. In Continuous Medical Education, the same pattern was identified, except for developmental course of childhood disorders, who appeared among the most referenced issues.

Aspects related to youth and relevant to transitional care were reported to be addressed during CAP training in 7/15 (47%) of responding countries (e.g., training providing with a global understanding of mental health services taking care of adolescents) to 11/15 of responding countries (73%) (e.g., treatments for adolescents and young adults specifically discussed in pharmacotherapy training) (Table 1).

#### Training Contents: Aspects of Cross-Training With AP

Having some theoretical training in AP along with CAP ***theoretical education*** was mandatory in 16/19 countries (84%), more specifically in 13/16 countries with specialization route in CAP (81%) and in 3/3 (100%) of generalist countries. In countries with a specialization route in CAP, the content of AP theoretical programme was different in AP education and in CAP education in 4/17 (24%) countries.

In CAP ***practical training***, placements in AP were mandatory in 17/19 countries (89%), more specifically in 13/15 (87%) countries with a CAP specialization and in 3/3 generalist countries (100%) (Table 2). In countries with specialization route in CAP, the mean number of mandatory placements in AP was 2,3 (12 respondents), for a mean total clinical experience of 14,6 months (12 respondents). In generalist countries, the mean mandatory number of placements in AP was 3 and mean total duration was 5 months.

#### Training Contents: Aspects of Common Background With AP

The mean level of adherence with UEMS recommendations of the CAP programmes was 8.3 (range 5–10) (Table 2).

Regarding the ***theoretical content*** recommended by the UEMS (Table 3), 20/23 topics were addressed during CAP training in more than two-third of countries. Old age, psychiatric aspects of public health and leadership, administration, management, economics were the least addressed, respectively in 5/13 countries (38%), 9/15 countries (60%) and 8/15 countries (53%).

As regards the ***practical training***, and the mandatory placements in disciplines other than AP during CAP training (Table 2), five disciplines were mandatory in association with CAP training in 47% (7/15) to 61% (11/18) of countries. The least frequent placements relevant for transition were intellectual disabilities (mandatory in 4/13 countries, 31%) and forensic (4/14, 29%). We observed the same pattern when countries with a specialization in CAP were considered.

#### Other Aspects of Training Contents

CAP was considered an independent speciality in 15/20 countries (75%). Most countries (except Belgium, Cyprus and Spain) had a national programme, implemented nationwide in 11/14 responding countries (79%).

There was a dominant theoretical orientation in CAP education in 8/17 countries (47%): biopsychosocial in 3/8 (37.5%), psychodynamic in 2/8 (25%), medical in 1/8 (12.5%) and bio-medical in 2/8 (25%).

## Discussion

Training of psychiatrists is identified as a determining factor in improving transitional planning and care, both from the perspectives of trainees and youth experiencing transition between services (12, 13, 21, 23). A more informed training and education in transition in both AP and CAP curriculum is likely to facilitate transition and to have long term-effects in terms of mental health prevention. About half of all lifetime cases of mental illness start by the age of 14, rendering prevention and early treatment among adolescents all the more necessary, and emphasizing the importance for CAP specialists to be informed about general psychiatry (37). Around one-fourth of patients in AP aged 18–25 had previously received care at CAP (38), while around one-third of late teenagers/young adults at CAP need care from AP in adulthood (39). This stresses the importance of a smooth transition instead of rigid boundaries between disciplines, since mental disorders in adolescence predict mental health problems in adulthood (40). Parents of children treated in CAP are more likely to have mental health problems themselves, while children of patients treated in AP will be more prone to mental health difficulties. Providing a safe environment to children, for example in developing parental skills, plays a crucial role in preventing mental health conditions among youth and should be emphasized in all training routes. Early detection of emergent mental disorders is another topic that highlights the importance for specialists in both CAP and AP to develop knowledge in core competencies of each specialty.

This article provides the results of a survey describing the current status of training in transitional care in both AP and CAP postgraduate training across Europe. We also report data on the respective content of AP and CAP postgraduate training, with a particular focus on the amount of theoretical and practical cross-training between both specialties.

### Transition Training and Developmental Topics in AP and CAP Training

#### During Adult Psychiatry Training

Previous findings showed that only 27% of trainees think that they have good knowledge in transitional care (21). Our results offer a potential explanation for this lack of knowledge of transition, as it is seldom a mandatory topic during AP training.

As regards issues related to transition, some progress is still required in the AP curriculum as they are addressed in only around half of the countries. For example, the AP-specific training programmes rarely address the issue of the key role of the family and related topics such as parent management techniques. Yet, the involvement of parents is essential in the early planning of transition (5) and at later stages in adult psychiatry, especially when patients are disabled and dependent on parental support.

Improved training in neurodevelopmental disorders, adolescent psychopathology and pharmacotherapy in the AP curriculum is also needed. Since intellectual disabilities do not disappear once a young person turns 18, the limited training on this issue in countries with AP specialization (theoretical training in 33% of countries [4/12] and placements in 9% [1/11]) is also an important gap. Training in this area for adult psychiatrists would allow a better consideration of this population and their specific needs in transition.

#### During Child and Adolescent Psychiatry Training

In the CAP curriculum, transition is addressed as a specific topic in 33% of countries (4/17) and most issues related to transition are addressed in more than 82% of the countries (9/11). However, these findings are not in line with the results from the above-mentioned study of Hendrickx et al. (21), in which CAP trainees from a larger sample, including 19 countries in common with ours, reported theoretical training in only 6 countries (16%). These differences between data from representatives of national associations and data from trainees suggest a possible gap between the national programmes and their implementation. In some countries, training in transition-relevant topics may also arrive at a late stage in the curriculum.

Transition is not a one-direction process sending young people from CAMHS to AMHS, but rather, is a continuous and closely connected care process based on shared decisions and planning between both services, involving young people and their caregivers as partners in decision-making early in the process. The development and implementation of guidelines (see www.uemscap.eu/working-group) to support transition of young people out of CAMHS would provide much needed guidance to clinicians about the transition process, options for transition pathways, and best practices in ensuring successful transitions (1, 36). These guidelines should be co-designed with youth, caregivers and clinicians, and be informed by the best available evidence. Such guidance would also be invaluable in helping those responsible for both AP and CAP curricula to ensure that all relevant topics are considered.

### Training Contents and Cross-Training

A recent scoping review identified 26 core components of successful CAMHS to AMHS transitions that could inform the basis for transitions pathways and guidelines (41). Among these components is the reciprocal core knowledge from both services that should be addressed through cross training as an essential component of optimal transitional care. The UEMS calls for a common trunk of core knowledge and skills in every national curriculum, including AP and developmental psychiatry, CAP, specific learning difficulties and intellectual disabilities. The high score of adherence of programmes to the UEMS recommendations (i.e., 8.2 in AP and 8.3/10 in CAP) is encouraging and the expected publication of specific UEMS recommendations for CAP is likely to improve responses to the specific needs of children, adolescents and young people during transition between services.

Collaborative working across services, involving joint meetings, communication, parallel working, as well as a shared cultural approach through education and training, are considered important for improving quality of transition and continuity of care (4, 27, 29). We tried to address these issues by exploring common topics and cross-training in the AP, CAP and generalist curricula.

Our data shows a joint theoretical base, but important variations in how this is actually addressed in training. Most topics recommended by the UEMS are mandatory in both AP and CAP training in the countries we studied. Some theoretical training in CAP is mandatorily associated with AP theoretical education in 18/19 countries (95%) and some theoretical training in AP is mandatorily associated with CAP theoretical education in 16/19 countries (84%). Among the other topics that we explored, emergency psychiatry, addiction, liaison and forensic are mandatory during AP training and CAP training in more than 63% of countries (12/19). However, this substantial joint theoretical base mostly concerns countries with a specialization route. In generalist countries, the common theoretical content is less comprehensive: AP, CAP and emergency psychiatry are the only three mandatory frequently addressed topics.

As opposed to theoretical training, practical cross-training in AP and CAP is not generalized: placements in AP are mandatorily associated with CAP training in 16/18 countries (89%) (in 13/15 countries with a specialization route in CAP, 87%); but placements in CAP are mandatorily associated with AP training in 14/20 countries (70%) (in 12/17 countries with a specialization route in AP, 71%). We found a higher proportion of mandatory placements in CAP within the AP-specialized curriculum than Oakley et al. (18) (i.e., 47%). Placements in AP within a CAP-specific route are similar to the 89% reported by Simmons et al. (20) and Barrett et al. (14), but this result is lower than the 100% reported by Oakley et al. (19). Therefore, it seems that placements in CAP have progressed in the AP curriculum, but placements in AP seem to have followed the opposite trend in the CAP curriculum. However, some caution is required in the comparison with the above-mentioned surveys: Oakley et al. (19) used a sample of trainees from 22 countries, with only 13 countries in common with ours; Simmons et al. (20) a sample of trainees from 28 countries, with only 17 countries in common; the survey of Barrett et al. (14) a sample of members of societies from 31 countries, with only 17 countries in common.

Furthermore, in contrast with our expectations, practical cross-training within both CAP as well as AP is not systematic within the generalist route, with CAP placements being mandatory in only 2/3 generalist countries (67%). Finally, our findings also suggest that mandatory placements in CAP during AP training are less numerous and represent a shorter practical experience in total than AP placements in CAP. However, these findings should be interpreted with caution due to the large proportion of missing information. The question of variable or limited exposure to CAP in AP training and, to a lesser degree, to AP in the CAP route was also raised elsewhere (14, 15, 19).

Concerns have been raised about the widespread specialization model across Europe which was described by Baessler et al. (22) and has been considered as a barrier to a common culture between CAP and AP (23). However, our findings about the adherence to UEMS standards highlight that separate specialities generally do provide a significant compulsory theoretical core cross-training, which is a facilitating factor for enhancing communication and information transfer. It is not clear if this theoretical cross-training is provided in joint sessions bringing together CAP and AP trainees, or in distinct groups. As regards the countries with a generalist curriculum, their adherence to UEMS standards is lower and offers no guarantee of practical cross-training.

Recommendations for improved training in transition care based on our findings are detailed in Table 4.

**Table 4:** Recommendations for improved training to transition care.

• Specific transition training modules on transition and related issues in postgraduate training curricula should be designed • In collaboration with international bodies (e.g., UEMS, European Federation of Psychiatry Trainees) and service users • In particular with young people of transition age, or with experience of moving from CAMHS to AMHS, to ensure that these modules cover all issues relevant to young people requiring transitional care (e.g., key role of the family, neurodevelopmental disorders, adolescent psychopathology or pharmacotherapy).
• Transition-focused continuing medical education and scientific communication should be developed.
• Theoretical and practical training content with input of both AP and CAP should be designed (i.e., joint faculty working groups), aiming at a common developmental approach (16, 42), particularly required for neurodevelopmental disorders like Attention-Deficit Hyperactivity Disorder (43) or Autism Spectrum Disorder (44). • Training modules on transition and adolescent development should be attended jointly by AP and CAP trainees.
• Implementation studies should be facilitated in order to assess the translation from theory into practice.
• Transition training should be extended to professionals in somatic health care, as transition also takes place between CAMHS or AMHS and somatic services. For example, the young people with autism have to deal with sleep disorders, epilepsy, gastrointestinal problems, respiratory, food and skin allergies that require specialized somatic care in addition to mental health care (45).

### Strengths and Limitations

This survey is one of the first to specifically address the question of transition as a specific topic in postgraduate training programmes and to explore the question of cross-training between AP and CAP in relation to this topic. The responses by representatives of faculty and national associations complement existing data from reviews of literature, accessible curricula, trainees, as well as youths and their families (13, 21, 23).

However, a number of limitations need to be pointed out. Our conclusions should be considered as preliminary as they are based on small numbers and only 54% of the representatives of solicited European countries returned the survey. Nevertheless, the answers came from the most populated countries. Some of the items yielded missing or unclear data, but this was of limited influence as they mostly concerned secondary issues. Finally, among the countries with their syllabus due to be revised soon, only partial information was received.

It should also be emphasized that this study was limited to training in psychiatry, whereas transition planning also involves many other professionals, for example, psychologists, social workers, and mental health nurses. Training in transition and related topics is thus relevant in other curricula. Future enquiry also needs to address interdisciplinary treatment collaboration and information transfer.

Finally, we did not assess specific modalities in our analysis of theoretical cross-training. For example, it seems important to know whether training was provided in joint sessions bringing together CAP and AP trainees or in distinct groups of trainees.

## Conclusion

This study suggests that specific training in transitional needs and management of patients in transitional care is still scarce across all training models. This may contribute to the well-recognized “transition gap” and should be addressed while designing best models of care and guidelines for transition. This situation calls for increased awareness of training gaps within psychiatry programmes and a need to enhance collaborative work and mutual understanding between CAP and AP services. A better postgraduate training on transition may thus proceed from programmes designed in joint faculty working groups and may take place through modules attended jointly by AP and CAP trainees. The role of those responsible for training programs at a local, national and international level to address the issue of transition and cross training between AP and CAP should also be considered. Finally, there is a need for further research on how best to develop specific training to transitional care that encompasses theoretical and practical knowledge which also includes strengthening collaborative teamwork, communication, information transfer and family involvement. Some further research should also be conducted on how this training would have an impact on the success of transitions experienced by young people. Apart from better clinicians, bridging the gap between services involves a change in research cooperation, transition planning, organization of care, mutual understanding about each other's workplaces, and bringing together different care cultures and different perspectives such as family-oriented care in CAP and individual-oriented care in AP.

## Data Availability Statement

The data that support the findings of this study are available on request to the authors at f-russet@chu-montpellier.fr.

## Ethics Statement

The survey is part of the MILESTONE project, for which an ethics approval was granted by the Ethics Committee Comité de protection des personnes Sud Méditerranée I. No further ethical approval was required for this specific survey, as no personal data were collected.

## The Milestone Consortium

Swaran Singh, Helena Tuomainen, Jason Madan, Jane Warwick, Cathy Street, Dieter Wolke, Moli Paul, Priya Tah, Rebecca Appleton, Alastair Canaway, James Griffin, Philip Wells, Rose-Marie Lomax (University of Warwick, UK), Giovanni de Girolamo, Giulia Signorini (Saint John of God Clinical Research Center, Italy), Paramala Santosh, Natalie Heaney, Mathilde Mastroianni, Laura Adams, Sagar-Ouriaghli I, Kate Lievesley, Jatinder Singh, Federico Fiori (Kings College London, UK), Diane Purper-Ouakil, Frédérick Russet, Virginie Maurice, Véronique Humbertclaude (Hôpital Saint Eloi, France), Athanasios Maras, Larissa van Bodegom, Mathilde Overbeek (Yulius Academie, the Netherlands), Ulrike Schulze, Melanie Saam, Ulrike Breuninger, Anne Sartor, Elena Tanase, Vehbi Sakar (University of Ulm, Germany), Sabine Tremmery, Gaëlle Hendrickx, Veronique De Roeck (KU Leuven, Belgium), Fiona McNicholas, Aleksandra Gronostaj, Ingrid Holme, Lesley O Hara (University College Dublin, Ireland), Tomislav Franić, Nikolina Davidović (University Hospital Split, Croatia), Frank Verhulst, Gwen Dieleman, Suzanne Gerritsen (Erasmus MC, The Netherlands), Kate Lievesley (HealthTracker, UK), Amanda Tuffrey, Anna Wilson, Charlotte Gatherer, Leanne Walker (Young project advisors, University of Warwick, UK), Andrea Wohner (concentris research management GmbH, Germany).

## Author Contributions

FR and VH were in charge of the study design, conducted the data search and analysis, and wrote the first draft of the manuscript. DP-O and ST were in charge of Work Package lead, study design, and writing. ND, GD, KD-Ć, TF, SG, GG, GH, HK, FM, AM, SP, MP, AS, US, CS, HT, DW, and SS provided substantial contribution to the final manuscript. All authors read and approved the final manuscript.

## Funding

The MILESTONE project received funding from the European Union's Seventh Framework Programme for research, technological development, and demonstration under Grant Agreement No. 602442. The European Commission (FP7 programme) had no involvement in the writing of the manuscript or in the decision to submit the manuscript for publication. SS was supported by the National Institute for Health Research (NIHR) Applied Research Centre (ARC) West Midlands.

## Author Disclaimer

The views expressed are those of the authors and not necessarily those of the NIHR or the Department of Health and Social Care. This Health Policy reflects only the author's views and the EU is not liable for any use that might be made of the information contained therein.

## Conflict of Interest

SP is employed by HealthTracker Ltd. The remaining authors declare that the research was conducted in the absence of any commercial or financial relationships that could be construed as a potential conflict of interest.

## Publisher's Note

All claims expressed in this article are solely those of the authors and do not necessarily represent those of their affiliated organizations, or those of the publisher, the editors and the reviewers. Any product that may be evaluated in this article, or claim that may be made by its manufacturer, is not guaranteed or endorsed by the publisher.

## Footnotes

^1^Number of countries/number of respondent countries.
