# Supplementary material for: Are Psychiatrists Trained to Address the Mental Health Needs of Young People Transitioning From Child to Adult Services? Insights From a European Survey
Source: Front Psychiatry. 2022 Feb 9;12:768206. doi: 10.3389/fpsyt.2021.768206 (PMC8864158; doi:10.3389/fpsyt.2021.768206)
Supplement: Supplementary file 1 [file Data_Sheet_1.docx]

**Supplementary material 1**

Synthesis of UEMS recommendations

The UEMS (European Union of Medical Specialists) established in 1962, is a collective of national associations of medical specialists in Europe, established in a voluntary capacity to safe guard and uphold the highest level of Continuing Medical Education, Post Graduate Training and Quality Assurance in doctors across Europe. In order to ensure free travel of specialists across Europe, patient safety and parity of clinical services, there was recognition of the need for European standardization of medical education and training such that all doctors would have the same core competencies.

In 2017, specific psychiatry-based recommendations were developed in the attempt to harmonize training across Europe. Recommendations were made as to the content and duration of training:

- The minimum duration was set at five years.

- A combination of theory and clinical practice was proposed, ensuring clinical exposure to psychiatric conditions throughout the life span, and across settings.

- Inclusion of knowledge transfer of medicine, and both theory and practical aspects of research were considered essential.

- Trainee’s role as teachers and leaders were recognised by an inclusion of exposure to teaching, advocacy and leadership roles, as was a mandatory weekly requirement for clinical and educational supervision.

- A ‘common trunk’ was considered the compulsory part of the educational input and shared by all trainees, irrespective of subsequent specialisation. This included specifications regarding the setting of the trainees’ experience: in-patient psychiatry, outpatient psychiatry, liaison, and emergency psychiatry, Psychotherapy. Working with different age groups and cohorts was also considered mandatory during this common trunk and should include general adult psychiatry, old age psychiatry, psychiatric aspects of substance misuse, developmental psychiatry (child and adolescent psychiatry, learning difficulties and mental handicap) and forensic psychiatry. Specialty training followed this common trunk, but was not further elaborated upon.
