# Supplementary material for: Are Psychiatrists Trained to Address the Mental Health Needs of Young People Transitioning From Child to Adult Services? Insights From a European Survey
Source: Front Psychiatry. 2022 Feb 9;12:768206. doi: 10.3389/fpsyt.2021.768206 (PMC8864158; doi:10.3389/fpsyt.2021.768206)
Supplement: Supplementary file 2 [file Data_Sheet_2.docx]

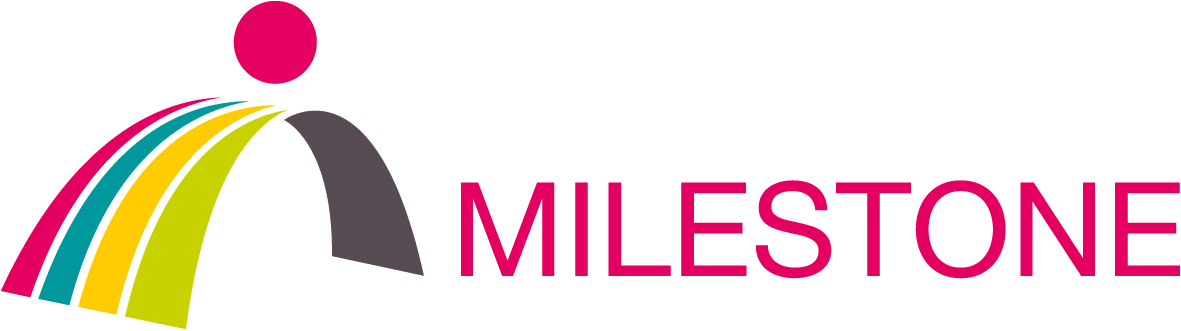
**SURVEY OF**

**SPECIALISTS' TRAINING**

**IN PSYCHIATRY IN EUROPE**

Transition to adulthood is the period of onset of some of the most serious mental disorders. Transition-related discontinuity of mental-health care is a major socioeconomic and societal challenge for the EU. The MILESTONE project is an EU-wide study determining care gaps in current services across diverse healthcare systems and evaluating an innovative transitional care model.

In the framework of this project, we aim to collect data about the training of professionals working in child/adolescent and adult mental health services across the 8 European countries participating in the MILESTONE project.

The following questionnaire is focused on psychiatrists' training in Europe and we would be very grateful if you could help us, through your answers, to build an overview of what currently exists.

The questionnaire is divided into 3 sections:

*- Part I. General questions about psychiatry training page 2*

*- Part II. Training and specialization in Adult Psychiatry page 8*

*- Part III. Training and specialization*

*in Child and Adolescent Psychiatry page 16*

Please identify yourself before starting :

* Name of the organization or the training centre:

………………………………………………………………………………………………….

………………………………………………………………………………………………….

* Location:

- country= - city=

* Name and role of the person(s) who completed the questionnaire:

………………………………………………………………………………………………….………………………………………………………………………………………………….

………………………………………………………………………………………………….

………………………………………………………………………………………………….

………………………………………………………………………………………………….………………………………………………………………………………………………….

***Part I. GENERAL QUESTIONS ABOUT PSYCHIATRY TRAINING***

**1.1.** After completing medical studies, is there a selection process *(e.g. exam, competitive examination, ranking…)* when applying to Adult Psychiatry or Child and adolescent psychiatry?

*Yes* □ *No* □

**1.1.a.** If YES, please state which kind : …………………………………………………………........

……………………………………………………………………………………………………………..

……………………………………………………………………………………………………………..

**1.2.** In your country, which is the main official training scheme in psychiatry?

- **A.** *One curriculum only, consisting in generalist training?* □

*(and optional complementary specialisations)*

- **B.** *Different specialisation routes, separate from the beginning?* □

*(monospecialities)*

- **C.** *Common trunk + advanced specialities?* □

**1.2.a.** Is this main official scheme existing in your country currently under review?

*Yes* □ *No* □

**1.2.b.** If YES, when is this revision expected to become effective?............................................

**1.2.c.** If YES, please provide details about the change to come in the official training scheme :

……………………………………………………………………………………………………………

……………………………………………………………………………………………………………

……………………………………………………………………………………………………………

……………………………………………………………………………………………………………

**1.3.** ***If your country belongs to group A (« Generalist training ») :***

How many years does it take to graduate as a specialist in psychiatry ?........................................

**1.4.** ***If your country belongs to group B or C (« Monospecialities » or " Common trunk + advanced specialities"):***

Which of the following specialties/subspecialties are official specialities and how long is the duration of training for each of them?

|  | *Official specialty?*  *Yes/No* | *If YES,*  *duration (in years)?* |
| --- | --- | --- |
| *- Adult psychiatry* |  |  |
| *- Child & Adolescent Psychiatry* |  |  |
| *- General psychiatry (*)* |  |  |
| *- Forensic Psychiatry* |  |  |
| *- Old age psychiatry* |  |  |
| *- Psychiatry of learning disabilities* |  |  |
| *- Handicap psychiatry* |  |  |
| *- Neuropsychiatry* |  |  |
| *- Addiction and substance misuse* |  |  |
| *- Other (Please provide details):*  *…………………………………………………*  *…………………………………………………*  *…………………………………………………*  *…………………………………………………*  *…………………………………………………*  *…………………………………………………* |  |  |

(*) If a speciality called "General psychiatry" exists in your country, please indicate what it consists of :

…………………………………………………………………………………………………………………….

…………………………………………………………………………………………………………………….

…………………………………………………………………………………………………………………….

…………………………………………………………………………………………………………………….

***THEORETICAL TRAINING***

**1.5.** In your country, is there an official syllabus programme for theoretical training in psychiatry?

*Please provide your answer in column A, B or C, according to the training scheme in your country. Groups B and C have been subdivided according to two specialties: Adult psychiatry and Child & Ado Psychiatry (CAP).*

|  | Group « A »  *Generalist training*  *Yes/No* | Group « B »  *Monospecialty*  *Adult psychiatry*  *Yes/No* | Group « B »  *Monospecialty*  *CAP*  *Yes/No* | Group « C »  *Adv Specialty*  *Adult Psychiatry*  *Yes/No* | Group « C »  *Adv Specialty*  *CAP*  *Yes/No* |
| --- | --- | --- | --- | --- | --- |
| *Official programme for theoretical training in psychiatry?* |  |  |  |  |  |

**1.5.a.** If YES, which subjects are mandatory parts of the theoretical training programme?

*Please provide your answer in column A, B or C, according to the training scheme in your country. Groups B and C have been subdivided according to two specialties: Adult psychiatry and Child & Ado Psychiatry (CAP).*

|  | Group « A »  *Generalist training*  *Yes/No* | Group « B »  *Monospecialty*  *Adult Psychiatry*  *Yes/No* | Group « B »  *Monospecialty*  *CAP*  *Yes/No* | Group « C »  *Adv Specialty*  *Adult Psychiatry*  *Yes/No* | Group « C »  *Adv Specialty*  *CAP*  *Yes/No* |
| --- | --- | --- | --- | --- | --- |
| *- Adult psychiatry* |  |  |  |  |  |
| *- Child & Ado*  *Psychiatry* |  |  |  |  |  |
| *- Old age* |  |  |  |  |  |
| *- Addiction and*  *substance misuse* |  |  |  |  |  |
| *- Forensic* |  |  |  |  |  |
| *- Emergency* |  |  |  |  |  |
| *- Liaison*  *psychiatry* |  |  |  |  |  |
| *- Mental handicap*  *and learning*  *disabilities* |  |  |  |  |  |
| *- Neurology* |  |  |  |  |  |
| *- Paediatrics* |  |  |  |  |  |
| *- Others (please*  *specify):*  *………………………………………………………………………...* |  |  |  |  |  |

**1.6.** In your country, is the involvement in research activities a mandatory part of the programme:

- in every specialty in psychiatry ? *Yes* □ *No* □

- if NO : - in adult psychiatry ? *Yes* □ *No* □

- in CAP ? *Yes* □ *No* □

**1.7.** In your country, has the concept of Evidence-Based Practice (EBP) been introduced in the training programme:

- in every specialty in psychiatry ? *Yes* □ *No* □

- if NO : - in adult psychiatry ? *Yes* □ *No* □

- in CAP ? *Yes* □ *No* □

***PRACTICAL TRAINING (CLINICAL PLACEMENTS)***

**1.8.** In your country, are clinical placements mandatory in psychiatry training?

*Please provide your answer in column A, B or C, according to the training scheme in your country. Groups B and C have been subdivided according to two specialties: Adult psychiatry and Child & Ado Psychiatry (CAP).*

|  | Group « A »  *Generalist training*  *Yes/No* | Group « B »  *Monospeciality*  *Adult psychiatry*  *Yes/No* | Group « B »  *Monospeciality*  *CAP*  *Yes/No* | Group « C »  *Adv Specialty*  *Adult Psychiatry*  *Yes/No* | Group « C »  *Adv Specialty*  *CAP*  *Yes/No* |
| --- | --- | --- | --- | --- | --- |
| *Mandatory clinical placements?* |  |  |  |  |  |

**1.9.** How many placements are mandatory in an entire training?

*Please provide your answer in column A, B or C, according to the training scheme in your country. Groups B and C have been subdivided according to two specialties: Adult psychiatry and Child & Ado Psychiatry (CAP).*

|  | Group « A »  *Generalist training*  *Yes/No* | Group « B »  *Monospeciality*  *Adult psychiatry*  *Yes/No* | Group « B »  *Monospeciality*  *CAP*  *Yes/No* | Group « C »  *Adv Specialty*  *Adult Psychiatry*  *Yes/No* | Group « C »  *Adv Specialty*  *CAP*  *Yes/No* |
| --- | --- | --- | --- | --- | --- |
| *How many mandatory placements in total ?* |  |  |  |  |  |

**1.10.** For each mandatory placement, do the trainees get access to :

- Educational supervision *^(*)^*? *Yes* □ *No* □

*(*) Educational supervision = trainee-oriented, consists of discussions with a tutor about clinical work, professional development, career advice, progress in acquiring competencies…(UEMS 2003)*

- Clinical supervision *^(**)^*? *Yes* □ *No* □

*(**) Clinical supervision = discussion with a senior professional about difficulties in the therapeutic relation between trainee and patient (Effenterre 2013)*.

**1.11.** Does a hospital or community setting have to comply with specific requirements to be able to take on a trainee? *Yes* □ *No* □

**1.11.a.** If YES, please specify which requirements:

……………………………………………………………………………...................................................

……………………………………………………………………………...................................................

……………………………………………………………………………...................................................

...........................................................................................................................................................

**1.12.** In which specialities are placements mandatory in psychiatry training?

*Please provide your answer in column A, B or C, according to the training scheme in your country. Groups B and C have been subdivided according to two specialties: Adult psychiatry and Child & Ado Psychiatry (CAP).*

|  | Group « A »  *Generalist training*  *Yes/No* | Group « B »  *Monospeciality*  *Adult psychiatry*  *Yes/No* | Group « B »  *Monospeciality*  *CAP*  *Yes/No* | Group « C »  *Adv Specialty*  *Adult Psychiatry*  *Yes/No* | Group « C »  *Adv Specialty*  *CAP*  *Yes/No* |
| --- | --- | --- | --- | --- | --- |
| *- Adult psychiatry* |  |  |  |  |  |
| *- Child & Ado*  *Psychiatry* |  |  |  |  |  |
| *- Old age* |  |  |  |  |  |
| *- Addiction and*  *substance misuse* |  |  |  |  |  |
| *- Forensic* |  |  |  |  |  |
| *- Emergency* |  |  |  |  |  |
| *- Liaison psychiatry* |  |  |  |  |  |
| *- Mental handicap*  *and learning*  *disabilities* |  |  |  |  |  |
| *- Neurology* |  |  |  |  |  |
| *- Paediatrics* |  |  |  |  |  |
| *- Others (please*  *specify):*  *………………………………………………………………………...* |  |  |  |  |  |

***CONTINUOUS MEDICAL EDUCATION***

**1.13.** In your country, is continuous medical education mandatory:

- in every specialty in psychiatry ? *Yes* □ *No* □

- if NO : - in adult psychiatry ? *Yes* □ *No* □

- in CAP ? *Yes* □ *No* □

**1.14.** Does the law require a regular recertification of psychiatrists? *Yes* □ *No* □

**1.14.a.** If YES, what are the requirements for recertification?

- minimum hours of *professional activity* ? *Yes* □ *No* □ How many? ………

- minimum hours of *continuous education* ? *Yes* □ *No* □ How many? ………

- Other ? Please specify : ……………………………………………………………………………

…………………………………………………………………………………………………………..

**1.14.b.** If YES, which professional/educational body is in charge of controlling recertification?

………………………………………………………………………………………………………………..

………………………………………………………………………………………………………………..

………………………………………………………………………………………………………………..

***You may have some further comments on general aspects of psychiatry training in your country:*** …………………………………………………………………………

………………………………………………………………………………………………………...

………………………………………………………………………………………………………...

………………………………………………………………………………………………………...

………………………………………………………………………………………………………...

………………………………………………………………………………………………………...

………………………………………………………………………………………………………...

………………………………………………………………………………………………………...

………………………………………………………………………………………………………...

………………………………………………………………………………………………………...

………………………………………………………………………………………………………...

………………………………………………………………………………………………………...

………………………………………………………………………………………………………...

………………………………………………………………………………………………………...

………………………………………………………………………………………………………...

………………………………………………………………………………………………………...

………………………………………………………………………………………………………...

………………………………………………………………………………………………………...

………………………………………………………………………………………………………...

………………………………………………………………………………………………………...

***Part II. TRAINING AND SPECIALIZATION in ADULT PSYCHIATRY***

**2.1.** In your country, what is the official title for Adult Psychiatry?

*- "Adult psychiatry"?* □ *- "Psychiatry"?* □

*- "General psychiatry"?* □ *- Other (please specify):…………………………….*

***PROGRAMME***

**2.2.** Is there a national programme for training in Adult Psychiatry? *Yes* □ *No* □

**2.2.a.** If YES, which professional/educational/governmental bodies are in charge of designing

it?....................................................................................................................................

…………………………………………………………………………………………………...

……………………………………………………………………………………………………

**2.3.** Is the implementation of this programme - nationwide? □

- dependent on region? □

- dependent on universities? □

**2.4.** According to you, does this programme comply with the standards recommended by the UEMS Board (section of psychiatry) and European Federation of Psychiatric Trainees (EFTP)? ^(*)^

*(*) Basic EFPT schema = training in in-patient psychiatry (short, medium and long stay), outpatient psychiatry (community psychiatry, day-hospital), liaison and consultation psychiatry, emergency psychiatry, psychotherapy ; in general adult psychiatry, old age psychiatry, psychiatric aspects of substance misuse, developmental psychiatry (chid and adolescents, handicap and learning disabilities)****,*** *and forensic psychiatry.*

1 5 10

/….…/……/……/……/……/.……/……/……/….../

*Not at all moderately completely*

**2.5.** In your country, is the training in adult psychiatry based:

*- only on theoretical training?*  *Yes* □ *No* □

*- only on practical training (placements)?* *Yes* □ *No* □

*- on both theoretical and practical training?* *Yes* □ *No* □

***THEORETICAL TRAINING***

**2.6.** What is the approximate *total* number of hours for theoretical training in Adult Psychiatry?

| *(please answer according to the training scheme(s) in your country)* | *How many hours?* |
| --- | --- |
| *- group A (generalist training)* |  |
| *- group B (for trainees who are in an adult psychiatry monospeciality)* |  |
| *- group C (for trainees who are in a common trunk + advanced specialty in adult psychiatry)* |  |

**2.7.** What are the teaching methods used for theoretical training in Adult Psychiatry (please indicate the average percentage of teaching time spent using them)? *(Multiple answers possible)*

*- lectures*  □ *…...% of total teaching time*

*- conferences – seminars*  □ *…...% of total teaching time*

*- interactive methods^(*)^* □ *……% of total teaching time*

*- other (please specify) :*…………………………………………………………………………………….

……………………………………………………………………………………………………………….

*(*) Literature review presentation, oral presentations by trainees, research or articles by the trainees …*

**2.8.** What is the mandatory content of theoretical training in Adult Psychiatry?

*(please tick the mandatory subjects)*

| *- psychopathology* |  | *- examination of a psychiatric patient* |  |
| --- | --- | --- | --- |
| *- diagnosis and classification* |  | *- psychological tests* |  |
| *- laboratory investigations* |  | *- specific disorders and syndromes* |  |
| *- developmental psychiatry* |  | *- old age psychiatry* |  |
| *- mental handicap* |  | *- psychotherapies* |  |
| *- psychiatric aspects of substance misuse* |  | *- social psychiatric interventions* |  |
| *- diversity in psychiatry* |  | *- community psychiatry* |  |
| *- legal, ethical and human rights issues in*  *psychiatry* |  | *- research methodology* |  |
| *- psychopharmacology* |  | *- psychiatric aspects of public health* |  |
| *- multidimensional clinical management* |  | *- emergency* |  |
| *- epidemiology of mental disorders* |  | *- other (please specify): :…………………………………………………*  *………………………………………………….*  *………………………………………………….* | |
| *- forensic* |  |  |  |
| *- leadership, administration, management,*  *economics* |  |  |  |

**2.9.** Is this mandatory content different when Adult Psychiatry theoretical training is provided in Child & Ado Psychiatry speciality?

*Yes* □ *No* □

**2.10.** If theoretical training in Child & Adolescent Psychiatry (CAP) is mandatory in Adult Psychiatry speciality training or in generalist training (cf. question 1.5.a), how many hours does it take in total for the whole of this training?

| *(please answer according to the training scheme(s) in your country)* | *How many hours of CAP theoretical education?* |
| --- | --- |
| *- group A (generalist training)* |  |
| *- group B (for trainees who are in an adult psychiatry monospeciality)* |  |
| *- group C (for trainees who are in a common trunk + advanced specialty in adult psychiatry)* |  |

**2.11.** In your country, is there one dominant theoretical orientation in Adult Psychiatry (e.g. psychodynamics, behavioural, family based, medical, bio-psycho-social, etc)?

*Yes* □ *No* □

**2.11.a.** If YES, which one:…………………………………………………………………………………...

***PRACTICAL TRAINING (CILNICAL PLACEMENTS)***

**2.12.** If placements in Adult Psychiatry (AP) are mandatory in Adult Psychiatry speciality training or in generalist training (cf. question 1.12.), what is the mandatory minimum duration of a placement?

| *(please answer according to the training scheme(s) in your country)* | *Mandatory minimum duration of a placement in AP (in months)* |
| --- | --- |
| *- group A (generalist training)* |  |
| *- group B (for trainees who are in an adult psychiatry monospeciality)* |  |
| *- group C (for trainees who are in a common trunk + advanced specialty in adult psychiatry)* |  |

**2.13.** If placements in Adult Psychiatry (AP) are mandatory in Adult Psychiatry speciality training or in generalist training (cf. question 1.12.), how many placements do the trainees have to do and for how many months in total?

| *(please answer according to the training scheme(s) in your country)* | *How many*  *mandatory placements in AP* | *For how many months in total* |
| --- | --- | --- |
| *- group A (generalist training)* |  |  |
| *- group B (for trainees who are in an Adult Psychiatry monospeciality)* |  |  |
| *- group C (for trainees who are in a common trunk + advanced specialty in AP)* |  |  |

**2.14.** If placements in Child & Adolescent Psychiatry (CAP) are mandatory in Adult Psychiatry speciality training or in generalist training (cf. question 1.12.), what is the mandatory minimum duration of a placement?

| *(please answer according to the training scheme(s) in your country)* | *Mandatory minimum duration of a placement in CAP (in months)* |
| --- | --- |
| *- group A (generalist training)* |  |
| *- group B (for trainees who are in an adult psychiatry monospeciality)* |  |
| *- group C (for trainees who are in a common trunk + advanced specialty in adult psychiatry)* |  |

**2.15.** If placements in Child & Adolescent Psychiatry (CAP) are mandatory in Adult Psychiatry speciality training or in generalist training (cf. question 1.12.), how many placements do the trainees have to do in this specialty and for how many months in total?

| *(please answer according to the training scheme(s) in your country)* | *How many*  *mandatory placements in CAP* | *For how many months in total* |
| --- | --- | --- |
| *- group A (generalist training)* |  |  |
| *- group B (for trainees who are in an Adult Psychiatry monospeciality)* |  |  |
| *- group C (for trainees who are in a common trunk + advanced specialty in AP)* |  |  |

**2.16.** In Adult Psychiatry, are placements in different types of settings mandatory (e.g. day hospital, medical hospital consultation, liaison, emergency, etc…)?

| *(please answer according to the training scheme(s) in your country)* | *Yes/No?* |
| --- | --- |
| *- group A (generalist training)* |  |
| *- group B (for trainees who are in an Adult Psychiatry monospeciality)* |  |
| *- group C (for trainees who are in a common trunk + advanced specialty in Adult Psychiatry)* |  |

**2.16.a.** If YES, in which of these settings are placements mandatory?

|  | Group « A »  *Generalist training*  *Yes/No?* | Group « B »  *Monospeciality*  *“Adult psychiatry”*  *Yes/No?* | Group « C »  *Adv Specialty*  *“Adult Psychiatry”*  *Yes/No?* |
| --- | --- | --- | --- |
| *- Psychiatric university hospital* |  |  |  |
| *- Psychiatric non-university hospital* |  |  |  |
| *- General (medical, surgical, psychiatry…)*  *hospital* |  |  |  |
| *- Private practice* |  |  |  |
| *- Community health centre* |  |  |  |
| *- In-patient long stay* |  |  |  |
| *- Day hospital* |  |  |  |
| *- Other (please specify):*  *……………………………………………….. …………………………………………………………………………………………* |  |  |  |

***TRAINING IN PSYCHOTHERAPY***

**2.17.** Is training in psychotherapy mandatory in Adult Psychiatry?

| *(please answer according to the training scheme(s) in your country)* | *Yes/No?* |
| --- | --- |
| *- group A (generalist training)* |  |
| *- group B (for trainees who are in an Adult Psychiatry monospeciality)* |  |
| *- group C (for trainees who are in a common trunk + advanced specialty in Adult Psychiatry)* |  |

**2.17.a.** If YES, does psychotherapy training have to be : *(Multiple answers possible)*

*- theoretical? Yes* □  *No* □ *For how many hours in total?......................*

*- practical? Yes* □  *No* □ *For how many hours in total?......................*

**2.18.** Which types of psychotherapy exist in Adult Psychiatry? Are they mandatory?

| *(please answer according to the training scheme in your country)* | Group « A »  *Generalist training*  *Yes/No* | Group « B »  *Monospeciality*  *“Adult psychiatry”*  *Yes/No* | Group « C »  *Adv Specialty*  *“Adult Psychiatry”*  *Yes/No* | *Mandatory?*  *Yes/No* |
| --- | --- | --- | --- | --- |
| *- Psychodynamics*  *(psychoanalysis)* |  |  |  |  |
| *- Cognitive Behavioural*  *Therapy* |  |  |  |  |
| *- Systemic – Family Therapy* |  |  |  |  |
| *- Individual psychoeducation* |  |  |  |  |
| *- Family psychoeducation* |  |  |  |  |
| *- Cognitive remediation* |  |  |  |  |
| *- Other (please specify) :*  *………………………………*  *……………………………*  *……………………………*  *……………………………* |  |  |  |  |

***ASSESSMENT METHODS***

**2.19.** Is trainees' work assessment mandatory in Adult Psychiatry?

| *(please answer according to the training scheme(s) in your country)* | *Yes/No?* |
| --- | --- |
| *- group A (generalist training)* |  |
| *- group B (for trainees who are in an Adult Psychiatry monospeciality)* |  |
| *- group C (for trainees who are in a common trunk + advanced specialty in Adult Psychiatry)* |  |

***If YES, please answer questions 2.20 to 2.22c.***

**2.20.** What is the object of the assessment? *- theoretical training* *Yes* □  *No* □

*(multiple answers possible)* *- placements* *Yes* □  *No* □

**2.21.** If YES to theoretical training assessment in Adult Psychiatry:

**2.21.a.** What is assessed? *- all subjects* □ *- only mandatory subjects* □

**2.21.b.** Which assessment methods are used? *- general exam about all subjects* □

*(multiple answers possible)* *- one exam for each subject* □

*- oral exam* □

*- written exam* □

*- multiple choice questions* □

*- other* *(please specify)*:…………………..

……………………………………..........

**2.21.c.** Are there consequences in case of poor assessment performance? *Yes* □ *No* □

If YES, please describe: ………………………………………………………………………..

……………………………………………………………………………………………………..

**2.22.** If YES to assessment of practical training in Adult Psychiatry:

**2.22.a.** Which placements are assessed?

*- every placement?* □

*- only the placements in mandatory disciplines?* □

**2.22.b.** Which assessment methods are used? *- Case presentations* □

*- Supervisor assessment* □

*- Portfolio* □

*- Other (please specify):……………………….......*

*………………………………………………........*

**2.22.c.** Are there consequences in case of poor assessment performance? *Yes* □ *No* □

If YES, please describe: ………………………………………………………………………..

……………………………………………………………………………………………………..

***Other QUESTIONS***

**2.23.** In your country, are there other official psychiatry training schemes apart from the main one identified in question 1.2?

*Yes* □ *No* □

**2.23.c.** If yes, please provide a short description: …………………………………………………….

………………………………………………………………………………………………………………

………………………………………………………………………………………………………………

**2.24.** Can you provide an estimate number of professionals trained through these schemes ?

………………………………………………………………………………………………………………………

***ABOUT “TRANSITION” IN ADULT PSYCHIATRY***

*In this questionnaire, the term "transition" is used to mean "health care transition" defined as a formal transfer of care from CAMHS to adult services, with the following optimal criteria (Singh, 2010) :*

*- a continuity of care ;*

*- and three further variables : period of parallel care (relational continuity), i.e. joint working ; transition planning meetings (cross-boundary and team continuity) ; optimal information transfer (information continuity).*

**2.25.** In your country, is “transition” a mandatory topic in the programme of Adult Psychiatry?

*Yes* □ *No* □

**2.25.a.** If NO, is it discussed in another teaching module during Adult Psychiatry training?

*Yes* □ *please specify: ……………………………………………… No* □

**2.26.** Whether it is discussed in Adult Psychiatry or another teaching module during Adult Psychiatry training, is it discussed through:

- some dedicated lectures ?  *Yes* □ *No* □

- case studies in placements? *Yes* □ *No* □

***More specifically …***

**2.27.** Are the following issues related to Transition discussed in Adult Psychiatry?

| (*Please indicate yes or no in the first column, and complete the table accordingly)* | *Yes/No* | *If YES, is it a mandatory subject?* | *If YES, through theoretical training?* | *If YES, through practical training?* |
| --- | --- | --- | --- | --- |
| *- developmental psychiatry* |  |  |  |  |
| *- developmental course of childhood disorders*  *in adolescents and young adults*  *(ADHD, Autism…)* |  |  |  |  |
| *- psychiatry and/or psychopathology*  *of adolescents* |  |  |  |  |
| *- psychometric assessment across*  *development (cognitive, neuropsychological,*  *adaptative…)* |  |  |  |  |
| *- key role of the family as a support to be used*  *and taken into account when taking care of*  *adolescents* |  |  |  |  |
| *- working with partners for an optimal care*  *of the adolescents* |  |  |  |  |

**2.28.** In Adult Psychiatry, are treatments for adolescents and young adults specifically discussed in pharmacotherapy training?

*Yes* □ *No* □

**2.29.** Is a placement in a hospital ward in charge of adolescents or young people mandatory during Adult Psychiatry training?

*Yes* □ *No* □

**2.30.** Does the training in Adult Psychiatry provide trainees with a global understanding of the CAMHS taking care of adolescents?

*Yes* □ *No* □

**2.31.** As far as you know, are the following issues related to Transition addressed in the continuing education of Adult Psychiatry in your country?

|  | *Yes/No* |
| --- | --- |
| *- developmental psychiatry* |  |
| *- developmental course of childhood disorders in adolescents and young adults (ADHD,*  *Autism…)* |  |
| *- psychiatry and/or psychopathology of adolescents* |  |
| *- psychometric assessment (cognitive, neuropsychological, adaptative…)* |  |
| *- key role of the family as a support to be used and taken into account when taking*  *care of adolescents* |  |
| *- working with partners for an optimal care of the adolescent* |  |

***You may have some further comments on Adult Psychiatry training and specialization in your country:***

………………………………………………………………………………………………………...

………………………………………………………………………………………………………...

………………………………………………………………………………………………………...

………………………………………………………………………………………………………...

………………………………………………………………………………………………………...

………………………………………………………………………………………………………...

………………………………………………………………………………………………………...

………………………………………………………………………………………………………...

………………………………………………………………………………………………………...

………………………………………………………………………………………………………...

………………………………………………………………………………………………………...

………………………………………………………………………………………………………...

………………………………………………………………………………………………………...

………………………………………………………………………………………………………...

………………………………………………………………………………………………………...

………………………………………………………………………………………………………...

………………………………………………………………………………………………………...

………………………………………………………………………………………………………...

………………………………………………………………………………………………………...

………………………………………………………………………………………………………...

***Part III. TRAINING AND SPECIALIZATION in CHILD & ADOLESCENT PSYCHIATRY***

**3.1.** Is Child & Ado Psychiatry an independent specialty in your country? *Yes* □ *No* □

**3.1.a.** If YES, since when?.........................................................................................................

**3.2.** Is Child & Ado Psychiatry specialization regulated by a legal dispositive in your country (minimum requirements for training, passing a specific exam etc…)?

*Yes* □ *No* □

***PROGRAMME***

**3.3.** Is there a national programme for training in Child & Ado Psychiatry?

*Yes* □ *No* □

**3.3.a.** If YES, which professional/educational/governmental bodies are in charge of designing it ?....................................................................................................................................

.................................................................................................................................

.................................................................................................................................

**3.4.** Is the implementation of this programme: - nationwide? □

- dependent on region? □

- dependent on universities? □

**3.6.** According to you, does this programme comply with the standards recommended by UEMS Board (section of psychiatry) and EFTP? (*) ?

*(*) In CAP a minimum of 5 years post-graduate training is required. A minimum of 3 years should be pure CAP training and experience should be gained both with a broad range of age groups and within varying settings of care. For the remaining training period, trainees should have the opportunity to gain basic clinical experience in related specialties : adult psychiatry / paediatric medicine / (ideally) paediatric neurology. All rotation periods should be of a minimum of 3 months duration.*

1 5 10

/….…/……/……/……/……/.……/……/……/….../

*Not at all moderately completely*

**3.7.** In your country, is training in Child & Ado Psychiatry based:

*- only on theoretical training?*  *Yes* □ *No* □

*- only on practical training (placements)?* *Yes* □ *No* □

*- on both theoretical and practical training?*  *Yes* □ *No* □

***THEORETICAL TRAINING***

**3.8.** What is the approximate *total* number of hours for theoretical training in Child & Ado Psychiatry?

| *(please answer according to the training scheme(s) in your country)* | *How many hours?* |
| --- | --- |
| *- group A (generalist training)* |  |
| *- group B (for trainees who are in a Child & Ado Psychiatry monospeciality)* |  |
| *- group C (for trainees who are in a common trunk + advanced specialty in Child & Ado Psychiatry)* |  |

**3.9.** Is theoretical training in Child & Ado Psychiatry mandatorily lectured by Child & Ado Psychiatry professors?

*Yes* □ *No* □

**3.10.** What are the teaching methods used for theoretical training in Child & Ado Psychiatry (please indicate the average percentage of teaching time spent using them)? *(Multiple answers possible)*

*- lectures*  □ *…...% of total teaching time*

*- conferences – seminars*  □ *…...% of total teaching time*

*- interactive methods^(*)^* □ *……% of total teaching time*

*- other (please specify) :*…………………………………………………………………………………….

……………………………………………………………………………………………………………….

*(*) Literature review presentation, oral presentations by trainees, research or articles by the trainees …*

**3.11.** What is the mandatory content of theoretical training in Child & Ado Psychiatry ?

*(please tick mandatory subjects)*

| *- psychopathology* |  | *- examination of a psychiatric patient* |  |
| --- | --- | --- | --- |
| *- diagnosis and classification* |  | *- psychological tests* |  |
| *- laboratory investigations* |  | *- specific disorders and syndromes* |  |
| *- developmental psychiatry* |  | *- old age psychiatry* |  |
| *- mental handicap* |  | *- psychotherapies* |  |
| *- psychiatric aspects of substance misuse* |  | *- social psychiatric interventions* |  |
| *- diversity in psychiatry* |  | *- community psychiatry* |  |
| *- legal, ethical and human rights issues in*  *psychiatry* |  | *- research methodology* |  |
| *- psychopharmacology* |  | *- psychiatric aspects of public health* |  |
| *- multidimensional clinical management* |  | *- emergency* |  |
| *- epidemiology of mental disorders* |  | *- other (please specify):*  *:……………………………….........................*  *………………………………………………….*  *………………………………………………….* | |
| *- forensic* |  |  |  |
| *- leadership, administration, management,*  *economics* |  |  |  |

**3.11.** Is this mandatory content different when Child & Ado Psychiatry theoretical training is provided in Adult Psychiatry speciality?

*Yes* □ *No* □

**3.12.** If theoretical training in *Adult psychiatry (AP)* is mandatory in Child & Ado Psychiatry (CAP) speciality training (cf. question 1.5.a), how many hours does it take in total for the whole of this training?

| *(please answer according to the training scheme(s) in your country)* | *How many hours of AP theoretical education in CAP speciality training?* |
| --- | --- |
| *- group A (generalist training)* |  |
| *- group B (for trainees who are in a Child & Ado Psychiatry monospeciality)* |  |
| *- group C (for trainees who are in a common trunk + advanced specialty in Child & Ado Psychiatry)* |  |

**3.13.** In your country, is there one dominant theoretical orientation in Child & Ado Psychiatry training (e.g. psychodynamics, behavioural, family based, medical etc)?

*Yes* □ *No* □

**3.13.a.** If YES, which one:..................................................................................................................

***PRACTICAL TRAINING (CLINICAL PLACEMENTS)***

**3.14.** If placements in Child & Ado Psychiatry (CAP) placements are mandatory in Child and ado Psychiatry speciality training or in generalist training (cf. question 1.12), what is the mandatory minimum duration of a placement?

| *(please answer according to the training scheme(s) in your country)* | *Mandatory minimum duration of a placement in CAP (in months)* |
| --- | --- |
| *- group "A" (generalist training)* |  |
| *- group B (for trainees who are in a Child & Ado Psychiatry monospeciality)* |  |
| *- group C (for trainees who are in a common trunk + advanced specialty in Child & Ado Psychiatry)* |  |

**3.15.** If placements in Child & Ado Psychiatry (CAP) placements are mandatory in Child & Ado Psychiatry speciality training or in generalist training (cf. question 1.12), how many placements do the trainees have to do and for how many months in total?

| *(please answer according to the training scheme(s) in your country)* | *How many*  *mandatory placements in CAP* | *For how many months in total* |
| --- | --- | --- |
| *- group A (generalist training)* |  |  |
| *- group B (for trainees who are in a Child & Ado Psychiatry monospeciality)* |  |  |
| *- group C (for trainees who are in a common trunk + advanced specialty in Child & Ado*  *Psychiatry)* |  |  |

**3.16.** If placements in Adult Psychiatry (AP) are mandatory in Child & Ado Psychiatry speciality training or in generalist training (cf. question 1.12), what is the mandatory minimum duration of a placement?

| *(please answer according to the training scheme(s) in your country)* | *Mandatory minimum duration of a placement in AP (in months)* |
| --- | --- |
| *- group "A"* *(generalist training)* |  |
| *- group B (for trainees who are in a Child & Ado Psychiatry monospeciality)* |  |
| *- group C (for trainees who are in a common trunk + advanced specialty in Child & Ado Psychiatry)* |  |

**3.17.** If placements in Adult Psychiatry (AP) are mandatory in Child & Ado Psychiatry speciality training or in generalist training (cf. question 1.12), how many placements do the trainees have to do in this speciality and for how many months in total?

| *(please answer according to the training scheme(s) in your country)* | *How many*  *mandatory placements*  *in AP* | *For how many months in total* |
| --- | --- | --- |
| *- group A (generalist training)* |  |  |
| *- group B (for trainees who are in a Child & Ado Psychiatry monospeciality)* |  |  |
| *- group C (for trainees who are in a common trunk + advanced specialty in*  *Child & Ado Psychiatry)* |  |  |

**3.18.** In Child & Ado Psychiatry training, are placements in different types of settings mandatory (day hospital, medical hospital consultation, liaison, emergency…)?

| *(please answer according to the training scheme(s) in your country)* | *Yes/No?* |
| --- | --- |
| *- group "A" (generalist training)* |  |
| *- group B (for trainees who are in a Child & Ado Psychiatry monospeciality)* |  |
| *- group C (for trainees who are in a common trunk + advanced specialty in Child & Ado Psychiatry)* |  |

**3.18.a.** If YES, in which ones:

| *(please answer according to the training scheme(s) in your country)* | Group « A »  *Generalist training*  *Yes/No?* | Group « B »  *Monospeciality*  *Child & Ado psychiatry*  *Yes/No?* | Group « C »  *Adv Specialty*  *Child & Ado*  *Psychiatry*  *Yes/No?* |
| --- | --- | --- | --- |
| *- Psychiatric university hospital* |  |  |  |
| *- Psychiatric non-university hospital* |  |  |  |
| *- General (medical, surgical, psychiatry…)*  *hospital* |  |  |  |
| *- Private practice* |  |  |  |
| *- Community health centre* |  |  |  |
| *- In-patient long stay* |  |  |  |
| *- Day hospital* |  |  |  |
| *- Other (please specify):*  *.............................................................*  *………………………………………………………………………………..............* |  |  |  |

***TRAINING IN PSYCHOTHERAPY***

**3.19.** Is training in psychotherapy mandatory in Child & Ado Psychiatry?

| *(please answer according to the training scheme(s) in your country)* | *Yes/No?* |
| --- | --- |
| *- group "A" (generalist training)* |  |
| *- group B (for trainees who are in a Child & Ado Psychiatry monospeciality)* |  |
| *- group C (for trainees who are in a common trunk + advanced specialty in Child & Ado Psychiatry)* |  |

**3.19.a.** If YES, does psychotherapy training have to be : *(multiple answers possible)*

*- theoretical? Yes* □  *No* □ *For how many hours in total?......................*

*- practical? Yes* □  *No* □ *For how many hours in total?......................*

**3.20.** Which types of psychotherapy exist in Child & Ado Psychiatry? Are they mandatory?

| *(please answer according to the training scheme(s) in your country)* | Group « A »  *Generalist training*  *Yes/No?* | Group « B »  *Monospeciality*  *Child & Ado psychiatry*  *Yes/No?* | Group « C »  *Adv Specialty*  *Child & Ado*  *Psychiatry*  *Yes/No?* | *Mandatory?*  *Yes/No* |
| --- | --- | --- | --- | --- |
| *- Psychodynamics*  *(psychoanalysis)* |  |  |  |  |
| *- Cognitive Behavioural*  *Therapy* |  |  |  |  |
| *- Systemic – Family Therapy* |  |  |  |  |
| *- Individual psychoeducation* |  |  |  |  |
| *- Family psychoeducation* |  |  |  |  |
| *- Cognitive remediation* |  |  |  |  |
| *- Other (please specify): …………..............................*  *…………………………….....*  *…………………………….....*  *............................................* |  |  |  |  |

***ASSESSMENT METHODS***

**3.21.** Is trainees' work assessment mandatory in Child & Ado Psychiatry?

| *(please answer according to the training scheme(s) in your country)* | *Yes/No?* |
| --- | --- |
| - *group "A" (generalist training)* |  |
| *- group B (for trainees who are in a Child & Ado Psychiatry monospeciality)* |  |
| *- group C (for trainees who are in a common trunk + advanced specialty in Child & Ado Psychiatry)* |  |

***If YES please respond to questions 3.22 to 3.24c***

**3.22.** What is the object of the assessment? *- theoretical training* *Yes* □  *No* □

*(multiple answers possible)* *- placements* *Yes* □  *No* □

**3.23.** If YES to theoretical training assessment in Child & Ado Psychiatry:

**3.23.a.** What is assessed? *- all subjects* □ *- only mandatory subjects* □

**3.23.b.** Which assessment methods are used? *- general exam about all subjects* □

*(multiple answers possible)* *- one exam for each subject* □

*- oral exam* □

*- written exam* □

*- multiple choice questions* □

*- other* *(please specify)*:…………………..

……………………………………........

**3.23.c.** Are there consequences in case of poor assessment performance? *Yes* □ *No* □

If YES, please describe: ………………………………………………………………………..

……………………………………………………………………………………………………..

**3.24.** If YES to assessment of practical training in Child & Ado Psychiatry:

**3.24.a.** Which placements are assessed?

*- every placement?* □

*- only the placements in mandatory disciplines?* □

**3.24.b.** Which assessment methods are used? *- Case presentations* □

*- Supervisor assessment* □

*- Portfolio* □

*- Other (please specify):……………………….....*

*………………………………………………......*

**3.24.c.** Are there consequences in case of poor assessment performance? *Yes* □ *No* □

If YES, please describe: ………………………………………………………………………....

……………………………………………………………………………………………………..

***LEGAL REQUIREMENTS FOR CAP PRACTICE***

**3.25. *If your country belongs to the A training scheme group ("generalist training"):***

Is a graduate in psychiatry allowed to work as a Child & ado Psychiatrist without any further qualification?

*Yes* □ *No* □

**3.26.** ***If your country belongs to the B or C training scheme group ("monospeciality" or "common trunk + advanced specialty"):***

Is a graduate in Adult Psychiatry allowed to work as a Child & Ado Psychiatrist?

*Yes* □  *No* □

***OTHER QUESTIONS***

**3.27.** ***If your country belongs to the A training scheme group ("generalist training"):***

Is there a subspecialisation or an official complementary training in Child & Ado Psychiatry during or after the general training?

*Yes* □ *No* □

**3.28.** ***Whatever your training scheme group:***

In your country, are there other official training schemes for Child & Ado Psychiatrists, apart from the main one?

*Yes* □ *No* □

**3.28.a.** If yes, please provide a short description? ……………………………………………………………………………………………………………….

……………………………………………………………………………………………………………….

**3.29.** Can you provide an estimate number of professionals trained through these others schemes?

………………………………………………………………………………………………………………………

***TRANSITION ISSUES IN CHILD AND ADOLESCENT PSYCHIATRY***

*In this questionnaire, the term "transition" is used for "mental health care transition" defined as a formal transfer of care from CAMHS to adult services, with the following optimal criteria (Singh, 2010):*

*- a continuity of care ;*

*- and three further variables : period of parallel care (relational continuity), i.e. joint working ; transition planning meetings (cross-boundary and team continuity) ; optimal information transfer (information continuity).*

**3.30.** In your country, is “transition” a mandatory topic in the programme of Child & Ado Psychiatry?

*Yes* □ *No* □

**3.30.a.** If NO, is it discussed in another teaching module during Child & Ado Psychiatry training?

*Yes* □ *please specify : ……………………………………………… No* □

**3.31.** Whether it is discussed in Child & Ado Psychiatry or in another teaching module during Child & Ado Psychiatry training, is it discussed through:

- some dedicated lectures ?  *Yes* □ *No* □

- case studies in placements? *Yes* □ *No* □

***More specifically***

**3.32.** Are the following issues related to Transition discussed in Child & Ado Psychiatry training?

| (*Please indicate yes or no in the first column, and complete the table accordingly*) | *Yes/No* | *If Yes, is it a mandatory subject?* | *If Yes, through theoretical training?* | *If Yes, through practical training?* |
| --- | --- | --- | --- | --- |
| *- developmental psychiatry* |  |  |  |  |
| *- developmental course of childhood disorders*  *in adolescents and young adults*  *(ADHD, Autism…)* |  |  |  |  |
| *- psychiatry and/or psychopathology*  *of adolescents* |  |  |  |  |
| *- psychometric assessment across*  *development (cognitive, neuropsychological,*  *adaptative…)* |  |  |  |  |
| *- key role of the family as a support to be used*  *and taken into account when taking care of*  *adolescents* |  |  |  |  |
| *- working with partners for an optimal care*  *of the adolescents* |  |  |  |  |

**3.33.** In Child & Ado Psychiatry training, is treatment for adolescents and young adults specifically discussed in pharmacotherapy training?

*Yes* □ *No* □

**3.34.** Is a placement in a hospital ward in charge of adolescents or young people mandatory during Child & Ado Psychiatry training?

*Yes* □ *No* □

**3.34.** Does the training in Child & Ado Psychiatry provide trainees with a global understanding of the AMHS taking care of adolescents and young adults?

*Yes* □ *No* □

**3.35.** As far as you know, are issues related to Transition addressed in the continuing education of Child & ado Psychiatry in your country?

|  | *Yes/No* |
| --- | --- |
| *- developmental psychiatry* |  |
| *- developmental course of childhood disorders in adolescents and young adults (ADHD,*  *Autism…)* |  |
| *- psychiatry and/or psychopathology of adolescents* |  |
| *- psychometric assessment (cognitive, neuropsychological, adaptative…)* |  |
| *- key role of the family as a support to be used and taken into account when taking*  *care of adolescents* |  |
| *- working with partners for an optimal care of the adolescent* |  |

***You may have some further comments on Child & Ado training and specialization in your country:***

………………………………………………………………………………………………………...

………………………………………………………………………………………………………...

………………………………………………………………………………………………………...

………………………………………………………………………………………………………...

………………………………………………………………………………………………………...

………………………………………………………………………………………………………...

………………………………………………………………………………………………………...

………………………………………………………………………………………………………...

………………………………………………………………………………………………………...

………………………………………………………………………………………………………...

………………………………………………………………………………………………………...

………………………………………………………………………………………………………...

………………………………………………………………………………………………………...

………………………………………………………………………………………………………...

………………………………………………………………………………………………………...

………………………………………………………………………………………………………...

………………………………………………………………………………………………………...

………………………………………………………………………………………………………...

………………………………………………………………………………………………………...

………………………………………………………………………………………………………...

*This is the end of the questionnaire.*

*Thank you very much for completing it.*

*Please send it back to the following email address:*

[*f-russet@chu-montpellier.fr*](mailto:f-russet@chu-montpellier.fr)
